# Early-Onset Diabetic E1-DN Mice Develop Albuminuria and Glomerular Injury Typical of Diabetic Nephropathy

**DOI:** 10.1155/2015/102969

**Published:** 2015-04-27

**Authors:** Mervi E. Hyvönen, Vincent Dumont, Jukka Tienari, Eero Lehtonen, Jarkko Ustinov, Marika Havana, Hannu Jalanko, Timo Otonkoski, Päivi J. Miettinen, Sanna Lehtonen

**Affiliations:** ^1^Department of Pathology, Haartman Institute, University of Helsinki, Haartmaninkatu 3, 00290 Helsinki, Finland; ^2^Children's Hospital, University of Helsinki, 00290 Helsinki, Finland; ^3^Department of Pathology, HUSLAB, Helsinki University Central Hospital, 05850 Hyvinkää, Finland; ^4^Department of Pathology, HUSLAB, Helsinki University Central Hospital, 00290 Helsinki, Finland; ^5^Laboratory Animal Centre, University of Helsinki, 00790 Helsinki, Finland; ^6^Biomedicum Stem Cell Center, University of Helsinki, 00290 Helsinki, Finland

## Abstract

The transgenic E1-DN mice express a kinase-negative epidermal growth factor receptor in their pancreatic islets and are diabetic from two weeks of age due to impaired postnatal growth of *β*-cell mass. Here, we characterize the development of hyperglycaemia-induced renal injury in the E1-DN mice. Homozygous mice showed increased albumin excretion rate (AER) at the age of 10 weeks; the albuminuria increased over time and correlated with blood glucose. Morphometric analysis of PAS-stained histological sections and electron microscopy images revealed mesangial expansion in homozygous E1-DN mice, and glomerular sclerosis was observed in the most hyperglycaemic mice. The albuminuric homozygous mice developed also other structural changes in the glomeruli, including thickening of the glomerular basement membrane and widening of podocyte foot processes that are typical for diabetic nephropathy. Increased apoptosis of podocytes was identified as one mechanism contributing to glomerular injury. In addition, nephrin expression was reduced in the podocytes of albuminuric homozygous E1-DN mice. Tubular changes included altered epithelial cell morphology and increased proliferation. In conclusion, hyperglycaemic E1-DN mice develop albuminuria and glomerular and tubular injury typical of human diabetic nephropathy and can serve as a new model to study the mechanisms leading to the development of diabetic nephropathy.

## 1. Introduction

Diabetic nephropathy affects every third patient with type 1 diabetes [1]. Advances in the management of diabetes may reduce or postpone the risk of renal complication [2], and, indeed, the incidence of nephropathy in type 1 diabetes has been reported to be declining [3]. Still, diabetic nephropathy is an important cause of morbidity in patients with diabetes and associates also with cardiovascular disease [4] and all-cause mortality [5]. The number of patients needing renal replacement therapy is rapidly increasing, mainly due to the global increase in the prevalence of type 2 diabetes [6, 7].

The first clinical sign of diabetic nephropathy is microalbuminuria [8], indicating an injury in the glomerular filtration barrier. The disease often progresses to macroalbuminuria (defined as urinary albumin excretion >300 mg/24 h) [9], and even nephrotic-range proteinuria is observed [10]. Subsequent decline in the glomerular filtration rate can lead to end-stage renal disease [11].

Pathological characteristics of diabetic nephropathy constitute predominantly of glomerular lesions [12]. Thickening of the glomerular basement membrane (GBM) is observed early in type 1 diabetes [13]. The most important pathologic feature is mesangial expansion, which is correlated to clinical findings of nephropathy [14] and to progression in albumin excretion [15]. The accumulation of mesangial matrix may eventually lead to nodular glomerular sclerosis, described first by Kimmelstiel and Wilson [16]. In advanced nephropathy, also tubular atrophy and interstitial fibrosis are observed [17].

Glomerular epithelial cells, podocytes, form the glomerular filtration barrier together with endothelial cells and the GBM. Both the structure and number of podocytes are affected in diabetes. Widening of podocyte foot processes, surrounding the glomerular capillaries, has been observed in renal biopsies of diabetic patients with increased albumin excretion [18, 19]. Reduced number of podocytes, reported in both type 1 [20] and type 2 diabetes [21], can result from podocyte detachment [22] or apoptosis [23].

The molecular pathways leading to albuminuria and the pathologic glomerular alterations are far from completely understood. There is an evident need for animal models for studies of the pathogenesis and the potential treatment strategies of diabetic nephropathy. The existing mouse models include induction of diabetes by streptozotocin [24], naturally mutated mouse lines like db/db [25] and Akita mice [26], and more recent genetically engineered models, for example, Ove26 mice [27] and endothelial nitric oxide synthase deficient diabetic mice [28]. Many of these models recapitulate the features of early nephropathy, various degrees of albuminuria and mild-to-moderate mesangial expansion, but rarely more advanced glomerulopathy [29, 30].

The homozygous transgenic mice expressing kinase-negative epidermal growth factor receptor (EGF-R) under the pancreatic duodenal homeobox-1 (pdx-1) promoter (E1-DN mice) are diabetic due to impaired postnatal growth of *β*-cell mass and subsequent reduced production of insulin [31]. Even the heterozygous E1-DN mice have impaired glucose tolerance in intraperitoneal glucose tolerance test [31]. The E1-DN mice are viable without insulin and can live up to one year. Thus, they can serve as a model to study the effects of long-term hyperglycaemia. The aim of this study was to characterize the development of renal injury in the diabetic E1-DN mice.

## 2. Materials and Methods

### 2.1. Animals

E1-DN mice in FVB background were generated previously [31]. The transgene comprised of a human kinase-deficient EGF-R cDNA with a myc-tag and a growth hormone polyA tail under mouse Pdx-1 promoter [31]. Mice were genotyped by dot blot analysis as previously described, and the homozygotes were identified based on blood glucose levels [31, 32]. Animals were maintained according to the principles of laboratory animal care, and the experiments were approved by the National Animal Experiment Board. Male E1-DN mice were examined, and male wild-type littermates were used as controls. Kidney samples were also collected from male nonlittermate controls of the same mouse strain in the same animal facility. During the one-year study period, mortality was 37.5% for the homozygous E1-DN mice, 12.5% for the heterozygous E1-DN mice, and 10% for the wild-type mice.

### 2.2. Blood Glucose and Urine Albumin Measurements

Random-fed blood glucose values were measured from tail vein with OneTouch Ultra Glucometer (Lifescan, Milpitas, CA, USA). Urine was collected as 24-hour samples in individual metabolic cages. The volume of urine was measured, and albumin concentration determined with mouse albumin ELISA kit (CellTrend, Luckenwalde, Germany).

### 2.3. Histology

Kidney samples were fixed in 10% formalin, dehydrated, and embedded in paraffin. Sections (5 *μ*m) were deparaffinised, stained with haematoxylin-eosin or periodic acid-Schiff (PAS) using standard procedures, and examined with Nikon Eclipse 800 microscope (Nikon Instruments Inc., Melville, NY, USA). Image-Pro Analyzer 6.0 (Media Cybernetics, Bethesda, MD, USA) software was used to measure the percentage of PAS-positive area in the glomerular tuft.

### 2.4. Immunohistochemistry

Immunoperoxidase staining was performed as previously described [33] using anti-activated caspase-3 (cleaved caspase-3, Asp175; Cell Signaling, Danvers, MA, USA) and anti-Ki-67 IgGs (Bethyl Laboratories, Montgomery, TX, USA) as primary antibodies, and VectaStain Elite ABC Kit (Vector Laboratories, Burlingame, CA) and 3-amino-9-ethylcarbazole (AEC) reagent (Sigma-Aldrich, St. Louis, MO, USA) for detection. Proliferation index was defined as the percentage of Ki-67 positive tubular cells and calculated from 100 microscope fields (approximately 10 000 cells) per mouse.

### 2.5. Immunofluorescence

Kidney samples were snap frozen in Tissue-Tek OCT-compound (Sakura, Alphen aan den Rijn, The Netherlands). Sections (5 *μ*m) were fixed with ice-cold acetone for 10 minutes, washed, blocked with CAS-block (Zymed, South San Francisco, CA, USA), and incubated with anti-nephrin GP-N2 (Progen Biotechnik GmbH, Heidelberg, Germany) and anti-activated caspase-3 (Cell Signaling) IgGs diluted in ChemMate (Dako, Glostrup, Denmark) and labeled with Alexafluor 488- and 555-conjugated secondary antibodies (Invitrogen, Carlsbad, CA, USA). After mounting in Moviol or Vectashield Mounting Medium (Vector Laboratories), the slides were analyzed using Leica SP2 confocal microscope (Leica Microsystems, Wetzlar, Germany). The staining intensity of nephrin was measured from a stack of five consecutive images using FIJI image analysis software and divided by the glomerular area.

### 2.6. Electron Microscopy

Kidney cortical samples were fixed in 2.5% glutaraldehyde in 0.1 M phosphate buffer (pH 7.2–7.4) at room temperature for 2 h, followed by postfixation in 1% osmium tetroxide for 2 h, stained en-bloc in 1% uranyl acetate in 10% ethanol for 1 h, dehydrated in ethanol, and embedded in LX-112 (Ladd Research Industries, Williston, VT). Thin sections were stained with uranyl acetate and lead citrate and examined in a JEM-1400 Transmission Electron Microscope (Jeol, Tokyo, Japan) equipped with Olympus-SIS Morada digital camera (Olympus Soft Imaging Solutions GmbH, Münster, Germany). The mesangial volume fraction, defined as mesangial cells, extracellular matrix, and the GBM, was calculated by morphometric analysis adapted from the method in [34], using the Fiji Software. The thickness of the GBM was measured from random capillary loops. Capillary loops with obvious bulging were excluded from the analysis to avoid overestimating the thickness of the GBM. The foot process width was determined as described in [35]. Shortly, the number of foot processes per capillary loop was counted, divided by the length of the GBM, and multiplied by *π*/4. The mesangial volume fraction and the foot process width are expressed as averages of measurements of three capillary loops per glomerulus.

### 2.7. Statistics

The data are presented as means ± standard error of mean (SEM), unless stated otherwise. Significance of the differences between the groups was evaluated by Student's unpaired *t*-test for normally distributed variables and by Mann-Whitney *U* test for nonnormally distributed variables or in case of a small sample size. For analyzing the correlation, Spearman's rho test was used. *P* < 0.05 was used as the limit for statistical significance. Analyses were performed using Excel (Microsoft, Redmond, WA, USA) or SPSS PASW Statistics (version 18) (IBM, Armonk, NY, USA) software.

## 3. Results

### 3.1. Diabetic E1-DN Mice Develop Albuminuria

**Figure 1:**
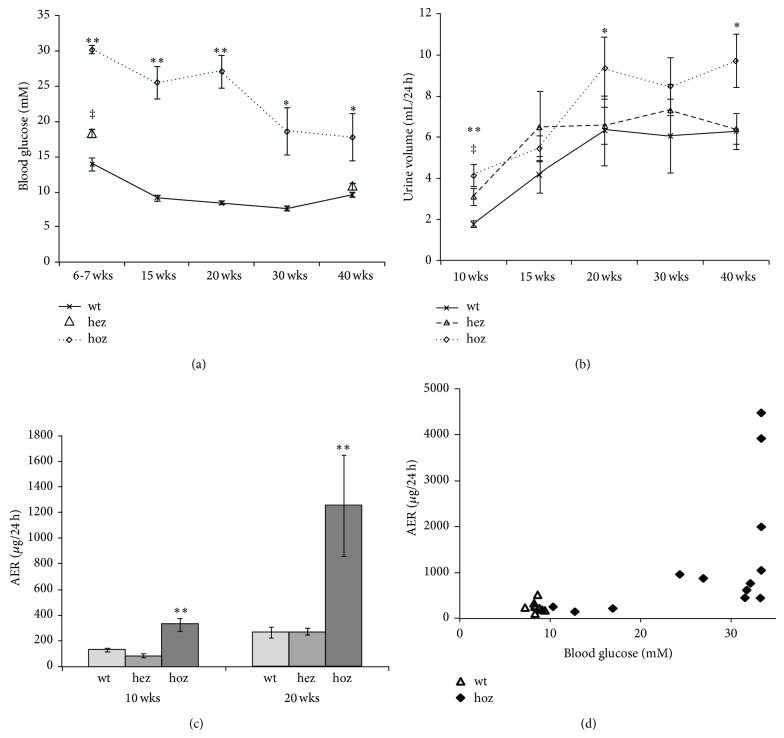
E1-DN mice develop hyperglycaemia and albuminuria. (a) Blood glucose is elevated in male E1-DN homozygous (hoz) mice (*n* = 8–16) compared to the wild-type (wt) mice (*n* = 6–10) at all the time points, ^**^ *P* < 0.01, ^*^ *P* < 0.05. At 6-7 weeks of age also the male E1-DN heterozygous (hez) mice (*n* = 14–23) are hyperglycaemic, ^‡^ *P* < 0.01, but at 40 weeks their blood glucose does not differ from the wt. (b) Urine volumes of the male E1-DN hoz (*n* = 9–16), hez (*n* = 7–16), and wt mice (*n* = 6–10). ^**^ *P* < 0.01 and ^*^ *P* < 0.05 for hoz versus wt, ^‡^ *P* < 0.01 for hez versus wt. (c) Increased albumin excretion rate (AER) is detected at both 10 and 20 weeks of age in the homozygous E1-DN male mice, ^**^ *P* < 0.01. Heterozygous mice do not develop albuminuria. In (a)–(c), data are shown as mean ± SEM. Mann-Whitney *U* test was used to compare groups. (d) AER is correlated to blood glucose at 20 weeks of age, *r* = 0.71, *P* < 0.001 (Spearman's rho), *n* = 22. As the upper limit of the glucometer is 33.3 mM, the samples giving the “high” code were analysed as 33.3 mM.

Monitoring of the blood glucose levels showed that the homozygous male E1-DN mice were overtly diabetic, as reported previously [31]. The blood glucose level was highest in young animals and stayed elevated during the whole follow-up (Figure 1(a)). The E1-DN heterozygous male mice were significantly hyperglycaemic at young age (Figure 1(a)), but at older age their blood glucose levels gradually decreased. Body weight did not differ significantly between the homozygous and wild-type mice at any time point (data not shown). Urinary albumin excretion and urine volumes were followed by 24-hour urine collections in metabolic cages. The E1-DN homozygous mice were polyuric when compared to the wild-type mice (Figure 1(b)). At young age also the heterozygous E1-DN mice had increased urine volumes (Figure 1(b)), consistent with the hyperglycaemia. An increase in albumin excretion rate (AER) in homozygous E1-DN mice was detected at the age of 10 weeks when compared to wild-type mice (Figure 1(c)). At 20 weeks some of the homozygous E1-DN mice developed massive albuminuria, in range of milligrams per 24 hours. Substantial variation was detected between individual mice in the homozygous E1-DN group; the mice with the highest blood glucose values developed the most severe albuminuria, and a strong correlation between the albumin excretion and blood glucose was evident at 20 weeks (*r* = 0.71, *P* < 0.001) (Figure 1(d)).

### 3.2. E1-DN Mice Develop Mesangial Expansion and Glomerular Sclerosis

**Figure 2:**
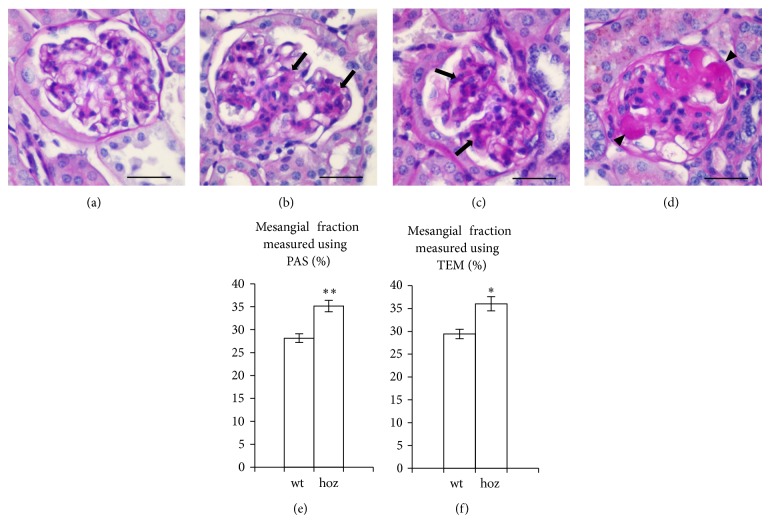
E1-DN mice present mesangial expansion and glomerular sclerosis. (a) Periodic acid-Schiff (PAS) staining of normal glomerulus of the wild-type mouse aged 50 weeks. ((b), (c)) Global mesangial expansion (arrows) is observed by PAS staining in the homozygous E1-DN mice aged 20 weeks (b) and 40 weeks (c). (d) Global nodular sclerosis (arrowheads) is seen in an E1-DN homozygous mouse aged 50 weeks. (e) The percentage of mesangial area in the glomerular tuft determined by morphometry of PAS-stained histological sections indicates an increase of 25% in the homozygous (hoz) E1-DN mice when compared to wild-type (wt) mice (*n* = 30 glomeruli from three 50 weeks old wild-type mice and *n* = 70 glomeruli from seven 20–50 weeks old homozygous mice). (f) Morphometric analysis performed on images obtained by transmission electron microscopy (TEM) confirms the increase of mesangial volume fraction by 22% in the glomeruli of E1-DN homozygous mice (*n* = 14 glomeruli from two 50 weeks old wild-type mice and *n* = 38 glomeruli from six 20–50 weeks old homozygous mice). In ((e), (f)), bars show mean ± SEM. ^**^ *P* < 0.01, ^*^ *P* < 0.05, Student's *t*-test. Scale bar: 25 *μ*m.

Mesangial expansion is a characteristic finding of diabetic glomerulopathy. Mesangial area, visualized by PAS staining, was found to be increased by 25% in the homozygous E1-DN mice when compared to the wild-type mice (35% versus 28%, *P* < 0.01, Students *t*-test, *n* = 30–70 glomeruli per group) (Figures 2(a)–2(e)). Electron microscopy confirmed an increase of 22% in the mesangial volume fraction in the homozygous E1-DN mice (36% versus 29%, *P* < 0.05, Students *t*-test, *n* = 14–38 glomeruli per group) (Figure 2(f)). In two homozygous E1-DN mice with the highest albumin excretion, the mesangial matrix accumulation was classified as focal, global nodular sclerosis by an expert pathologist who examined the samples blinded from the genotypes.

### 3.3. Albuminuric E1-DN Mice Exhibit Tubular Changes

**Figure 3:**
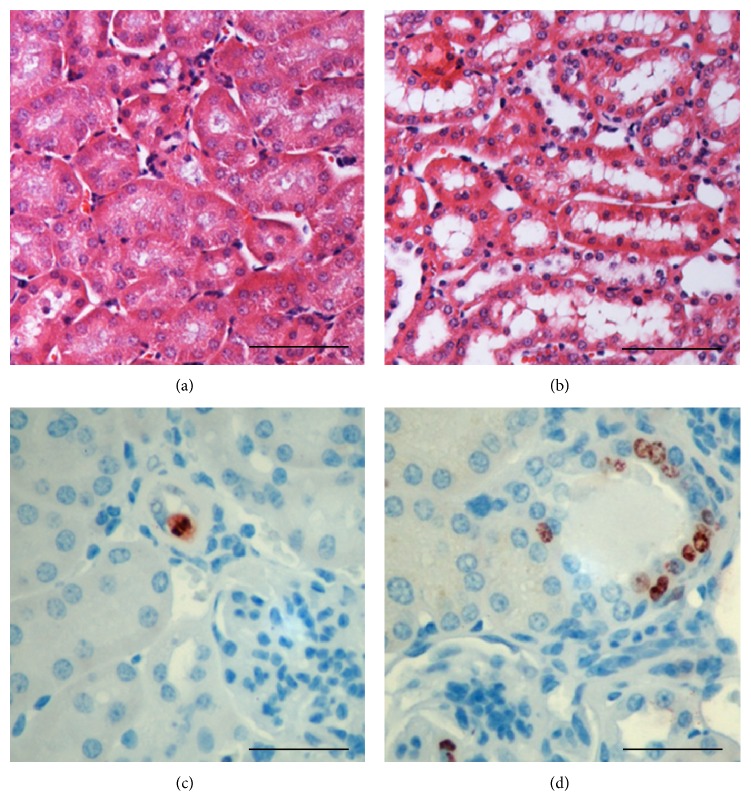
Renal tubules of E1-DN mice show morphological changes and increased proliferation. ((a), (b)) Hematoxylin and eosin staining shows flattened tubular epithelial cells in the albuminuric E1-DN homozygous mice (b) when compared to normal tall cuboidal epithelium in the wild-type mice (a). ((c), (d)) Immunostaining for Ki-67 reveals only occasional proliferating cells in the wild-type mice (c), whereas in the albuminuric E1-DN homozygous mice groups of proliferating cells are observed (d). Scale bar: 50 *μ*m ((a), (b)); 25 *μ*m ((c), (d)).

Flattened tubular epithelial cells and dilated tubular lumens were observed in the kidneys of albuminuric homozygous E1-DN mice in which the albumin excretion exceeded 1000 *μ*g/24 h at 20 weeks of age (Figures 3(a) and 3(b)). To study the tubular injury further, we stained proliferating cells with an antibody for Ki-67 (Figures 3(c) and 3(d)). Significantly increased tubular proliferation was observed in the albuminuric E1-DN mice (proliferation index 0.70%), compared to the control mice in which only sporadic proliferating cells were detected (proliferation index 0.18%) (*P* < 0.01, Student's *t*-test, *n* = 3-4 mice per group).

### 3.4. Podocyte Apoptosis Is Increased in E1-DN Mice

**Figure 4:**
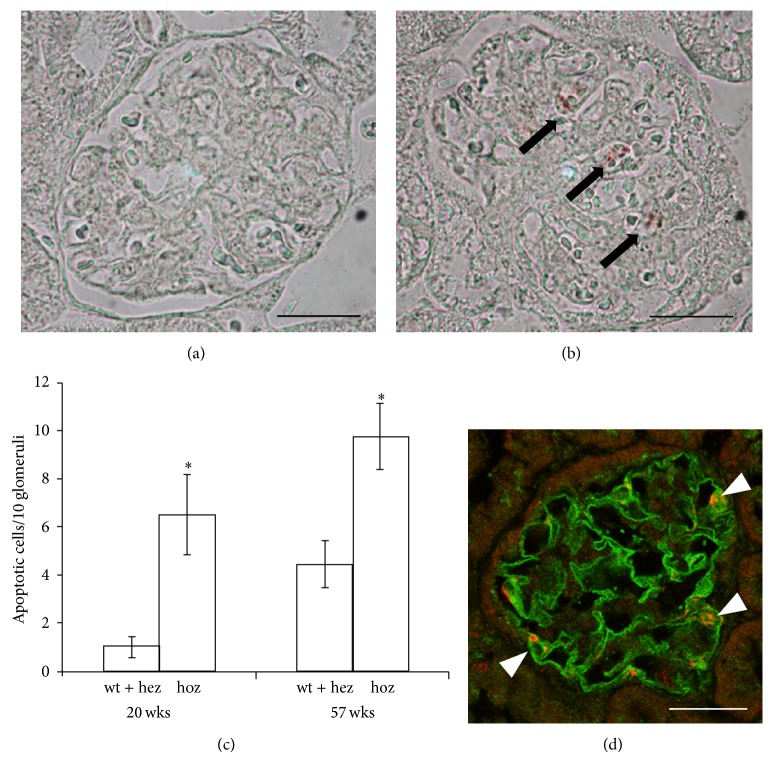
E1-DN mice show increased podocyte apoptosis. ((a), (b)) Staining for cleaved caspase-3 was used to identify apoptotic cells (arrows) in the glomeruli of wild-type (a) and homozygous E1-DN mice (b). (c) Apoptosis of glomerular cells is detected more often in the homozygous (hoz) E1-DN mice when compared to heterozygous (hez) and wild-type (wt) mice. Bars show the mean number of apoptotic cells per 10 glomeruli ± SEM, *n* = 4–8 mice/group. ^*^ *P* < 0.02, Mann-Whitney *U* test. (d) Confocal microscopy of kidney sections of 20 weeks old homozygous E1-DN mice stained for nephrin (green) and cleaved caspase-3 (red) indicates that the apoptotic cells in the glomeruli are podocytes. Scale bar: 25 *μ*m.

To study whether apoptosis plays a role in the glomerular injury in the E1-DN mice, apoptotic glomerular cells were stained with an antibody for activated caspase-3. Apoptotic cells were counted from 10 glomeruli from each mouse (*n* = 4–8 mice per group) by two researchers independently. Increased number of apoptotic cells was detected in the glomeruli of E1-DN homozygous mice when compared to wild-type and heterozygous E1-DN mice both at the age of 20 weeks (6.5 versus 1.0 apoptotic cells per 10 glomeruli, *P* < 0.02, Mann-Whitney *U* test), and in the oldest age group of 56-57 weeks (9.8 versus 4.4 apoptotic cells per 10 glomeruli, *P* < 0.02, Mann-Whitney *U* test) (Figures 4(a)–4(c)), although the amount of apoptotic glomerular cells was sparse in all mice. To characterize the nature of the apoptotic glomerular cells, we performed a double labelling for cleaved caspase-3 and nephrin revealing that the apoptotic cells were podocytes (Figure 4(d)).

### 3.5. Nephrin Expression Is Reduced in Diabetic E1-DN Mice

**Figure 5:**
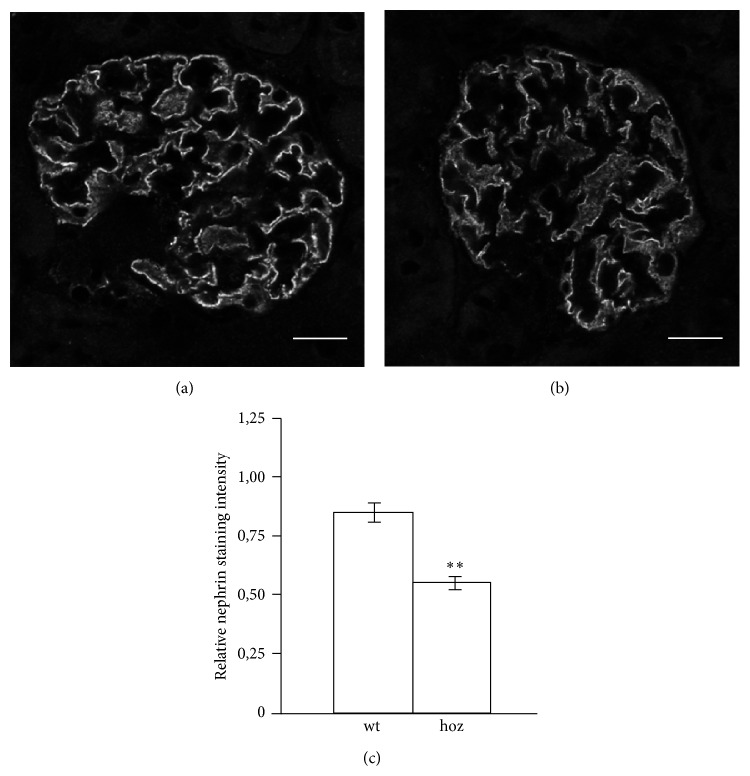
Expression of nephrin is reduced in the glomeruli of E1-DN mice. ((a), (b)) Confocal microscopy of wild-type (wt) (a) and homozygous (hoz) (b) E1-DN mouse kidney sections stained for nephrin. (c) Quantification of the expression level of nephrin in two wild-type mice and three albuminuric homozygous mice, five glomeruli per mice, reveals that the expression of nephrin is significantly lower in the E1-DN homozygous mice. Bars show mean ± SEM. ^**^ *P* < 0.01, Student's *t*-test. Scale bar: 20 *μ*m.

The expression of nephrin, the key molecule of the slit diaphragm structure connecting the podocyte foot processes, was studied by immunofluorescence staining and confocal microscopy (Figures 5(a) and 5(b)). Measurement of nephrin staining intensity indicated lower expression level in albuminuric homozygous E1-DN mice when compared to wild-type mice (Figure 5(c)). The expression patterns of other podocyte proteins examined, podocin and ZO-1, were not altered (data not shown).

### 3.6. E1-DN Mice Develop Glomerular Basement Membrane Thickening and Podocyte Foot Process Widening

**Figure 6:**
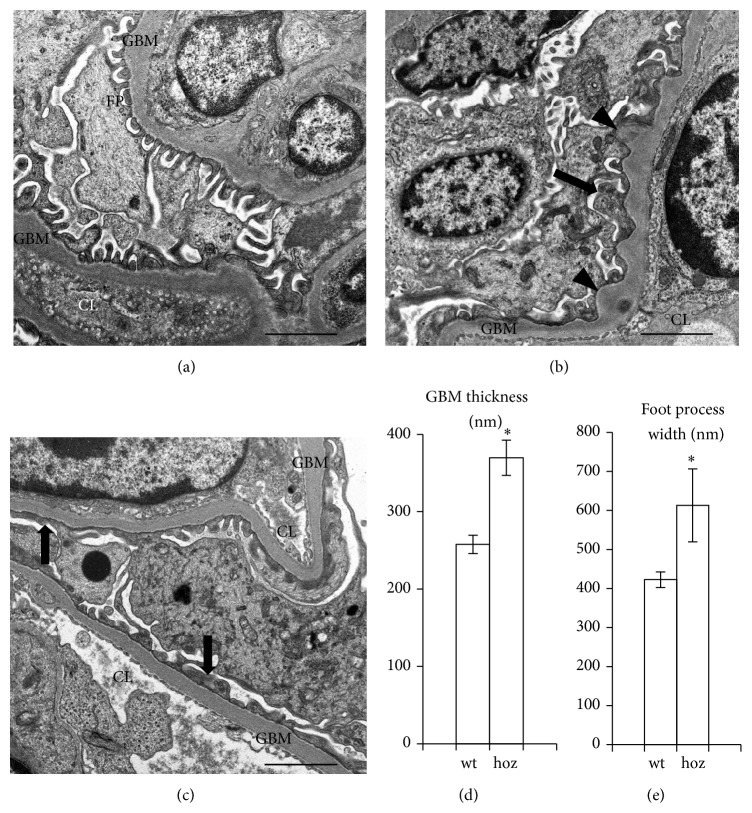
Electron microscopy indicates glomerular basement membrane thickening and podocyte foot process effacement in E1-DN mice. (a) In wild-type mouse (age 50 weeks) podocyte foot processes (FP) line regularly the glomerular basement membrane (GBM) around the capillary loops (CL). (b) In the E1-DN homozygous mouse (age 40 weeks) irregular thickening of the GBM (arrowheads) and podocyte foot process widening (arrow) are observed. (c) Foot process widening (arrows) is observed in 50 weeks old E1-DN homozygous mouse. (d) GBM is thicker in the E1-DN homozygous (hoz) mice (age 40–50 weeks) compared to the wild-type (wt) mice (age 50 weeks). The thickness of the GBM was calculated from three glomeruli in two wild-type and eight glomeruli in three homozygous E1-DN mice. (e) Podocyte foot processes are wider in the homozygous E1-DN mice (40–50 weeks of age) compared to the wild-type mice (50 weeks of age). Foot process width was calculated from 10 glomeruli in two wild-type and 9 glomeruli in three homozygous E1-DN mice. Bars show mean ± SEM. ^*^ *P* < 0.025, Mann-Whitney *U* test. Scale bar: 2 *μ*m.

Electron microscopic analysis of kidneys from two wild-type mice (age 50 weeks) and three homozygous E1-DN mice (age 40–50 weeks) revealed irregular thickening and bulging of the GBM and widening of the podocyte foot processes in the homozygous E1-DN mice (Figures 6(a)–6(c)). The width of the GBM was measured from random capillary loops in areas where the basement membrane was regularly shaped and foot processes appeared structurally normal and was found to be 43% higher in the E1-DN homozygous mice when compared to wild-type mice (258 nm versus 370 nm, *P* = 0.025, Mann-Whitney *U* test, *n* = 3–8 glomeruli per group) (Figure 6(d)). Podocyte foot processes were also 45% wider in these hyperglycaemic and albuminuric E1-DN mice when compared to wild-type mice (423 nm versus 613 nm, *P* = 0.013, Mann-Whitney *U* test, *n* = 9-10 glomeruli per group) (Figure 6(e)).

## 4. Discussion

This study describes the development of albuminuria in relation to hyperglycaemia in the diabetic E1-DN mice, a transgenic mouse model expressing kinase-negative EGF-R in pancreatic islets [31], and characterizes the pathologic changes in glomeruli including mesangial expansion and glomerular sclerosis, thickening of the GBM and widening of foot processes in albuminuric mice. Increased apoptosis was identified as one mechanism contributing to glomerular injury. In addition to glomerular changes, the E1-DN mice showed flattened tubular epithelium with dilated lumens and increased proliferation of tubular cells.

The kinase-negative EGF-R is expressed in the *β*-cells of the pancreatic islets, and it functions in a dominant negative manner, reducing phosphorylation of the endogenous EGF-R in response to EGF family ligands, resulting in a significantly reduced *β*-cell mass, low insulin production and subsequently high blood glucose, and early-onset diabetes [31]. The renal phenotype we now report is purely secondary to the long-acting hyperglycaemia. As blood glucose levels were higher in male E1-DN mice when compared to females, and male mice are generally more prone to renal injury, only male mice were examined in this study.

The homozygous E1-DN mice developed substantial albuminuria. The mean AER was 2.5-fold higher in the homozygous E1-DN mice compared to the wild-type littermates already at the age of 10 weeks and nearly five-fold higher at 20 weeks. The most severely affected mice had over 10-fold increase in AER. The individual variation in the levels of albuminuria can be explained by the variation in the blood glucose levels, as AER and blood glucose correlated at the age of 20 weeks. This reflects also the human diabetes, where hyperglycaemia is the main risk factor for diabetic nephropathy, as shown in several clinical studies [36, 37]. Intracellular excess of glucose is known to result in the formation of reactive oxygen species, which in turn cause inflammation and formation of advanced glycosylation end products [38].

Histological analyses revealed mesangial expansion and tubular changes in diabetic E1-DN mice. Mesangial expansion is an important factor in the progression of diabetic nephropathy [15], and mainly due to mesangial matrix accumulation, which can eventually lead to formation of nodular sclerosis, called Kimmelstiel-Wilson nodules [16]. The most severely affected E1-DN mice developed glomerular sclerosis resembling human diabetic glomerulopathy. Also tubular changes belong to the histological features of human diabetic nephropathy [12] and have been shown to predict disease progression in patients with type 2 diabetes and overt proteinuria [39]. Increased proliferation of tubular cells, possibly representing an adaptive mechanism to injury, has previously been observed to contribute to the renal enlargement in streptozotocin-induced diabetes in rats [40] and has been detected also in db/db mice [41]. The data is in line with the results of this study, showing increased proliferation in the tubules of the albuminuric E1-DN mice.

Increased glomerular apoptosis has previously been observed in kidney samples of human patients with diabetes [23] and streptozotocin-treated diabetic rats [42]. Further, reactive oxygen species have been shown to mediate podocyte apoptosis in the db/db mouse model [43]. We observed an increased number of apoptotic cells in the glomeruli of homozygous E1-DN mice and found that the apoptotic cells were podocytes. Apoptosis was rare in both diabetic and control mice, but as podocytes are terminally differentiated cells, even a small increase in the rate of apoptosis could affect the development of glomerular injury. Another mechanism for podocyte loss in diabetes is detachment. A recent study [22] described severe disruption and detachment of podocyte foot processes in patients with type 1 diabetes, leading to denuded areas of GBM, previously described in focal segmental glomerulosclerosis [44], and considered as part of the process leading to glomerular sclerosis [45]. In the E1-DN mice, no denuded areas of GBM were observed. However, podocyte foot process widening, a known feature of diabetic nephropathy [18, 19], was evident in the most albuminuric E1-DN mice. In addition, structural analysis by electron microscopy revealed thickening of the GBM, which is a well-documented and early finding in human diabetic nephropathy [13, 15]. The measurements of the thickness of the GBM were performed on areas where the GBM was of regular shape, but in addition, areas of irregular bulging of the membrane were observed.

The expression of nephrin was found to be decreased in the glomeruli of the albuminuric E1-DN mice, consistent with previous results obtained with the streptozotocin mouse model [46]. Also in human diabetic nephropathy the expression of nephrin protein [47] and mRNA [48] have been reported to be reduced and inversely correlated with the degree of proteinuria [48]. Whether this is causative or secondary to the disease progression and podocyte injury remains to be investigated. However, it has been suggested that the downregulation of nephrin is a selective change, as the expression of podocin, another podocyte protein, has been reported to remain unchanged in diabetes in humans [49], as well as in a diabetic mouse model [46]. Consistent with that, the expression of podocin and ZO-1 were not altered in immunofluorescence stainings of the E1-DN mouse glomeruli.

When compared to other mouse models of diabetes, E1-DN mice develop substantial albuminuria and glomerular pathology. It is notable that the renal changes are caused by hyperglycaemia instead of direct effects of the EGF-R transgene, and thus the E1-DN mouse model mimics human diabetes with poor glycaemic control. In addition, there is no need to consider the possible toxic effects on kidney, like in one of the most widely used murine models of diabetes, streptozotocin-induced destruction of pancreatic *β*-cells. The other widely used model, the db/db mouse, is insulin resistant and diabetic due to a mutation in the leptin receptor gene. Db/db mice serve as a good model for type 2 diabetes and albuminuria but do not develop severe glomerulopathy [25]. E1-DN mice are hypoinsulinemic [31] and show similarities with the OVE26 and Akita mice. The OVE26 is a promising mouse model of diabetic renal disease, overexpressing calmodulin in the pancreatic *β*-cells in mice in FVB background, and presenting high albuminuria, as well as severe glomerular pathology [27]. The Akita mice have a spontaneous mutation in insulin 2 gene and were originally described to have a modest renal phenotype in C57BL/6 strain [50]. When Akita mice were crossed to FVB/NJ background, they, however, developed high proteinuria and moderate mesangial expansion [26]. Recent studies thus indicate that the background strain of the mouse model is extremely important for the development of renal injury and that the FVB background is susceptible for renal disease. The FVB background may contribute to the relatively severe phenotype of the E1-DN mice.

One disadvantage of the E1-DN model is the large phenotypic variation between the individual mice, although the intensity of the renal phenotype is directly correlated to the blood glucose levels. Thus, designing an intervention study with the E1-DN mouse model would require a large number of mice. Histological and structural changes develop relatively late; the most severe changes reported here were detected at the age of 50 weeks. This is common for many of the mouse models and not surprising, considering that overt human diabetic nephropathy usually develops after 15 to 20 years of diabetes duration.

## 5. Conclusions

The hyperglycaemic E1-DN mice develop substantial albuminuria and histological and structural changes including mesangial expansion, thickening of the GBM, podocyte foot process widening, and altered tubular epithelial cell morphology and proliferation. Reduced expression of nephrin and increased apoptosis of podocytes might contribute to the development of glomerular injury. Altogether, these changes are typical of human diabetic nephropathy, and the E1-DN mice can serve as a good model to study the pathogenesis of the diabetic renal disease.
